# Continuous High Positive-End Expiratory Pressure May Worsen Renal Function in Patients With Acute Respiratory Distress Syndrome: Retrospective Analyses of a Nationwide, Multicenter Observational Study in Japan

**DOI:** 10.7759/cureus.35233

**Published:** 2023-02-20

**Authors:** Kazuto Yokoyama, Tadashi Kaneko, Yohei Ieki, Asami Ito, Eiji Kawamoto, Kei Suzuki, Ken Ishikura, Hiroshi Imai

**Affiliations:** 1 Emergency and Critical Care Center, Mie University Hospital, Tsu, JPN

**Keywords:** sofa score, apache ii score, acute kidney injury, acute respiratory distress syndrome, positive end expiratory pressure

## Abstract

Background: Positive end-expiratory pressure (PEEP), especially continuous high PEEP, is thought to be a risk factor for worsening renal function (WRF) due to impaired venous return and the development of renal interstitial edema. In this study, we investigated whether PEEP is a risk factor for WRF in patients with acute respiratory distress syndrome (ARDS), a representative pathology that requires continuous high PEEP for respiratory management.

Methods: We performed retrospective sub-analyses of the Japanese Association for Acute Medicine, a nationwide prospective observational registry of ARDS (FORECAST ARDS registry) prospective multicenter cohort study. WRF was defined on the basis of a worsening renal Sequential Organ Failure Assessment (SOFA) score. We performed univariate and multivariable analyses to identify possible risk factors for WRF, and propensity score analyses to compare the frequency of WRF according to cutoff values for the difference in PEEP between day 1 and day 4.

Results: We analyzed 151 cases. Multivariable analysis showed that the difference in PEEP (odds ratio (OR) 1.123 (95% confidence interval (CI) 1.017-1.240), *P* = 0.022) and male sex (OR 3.287 (95% CI 1.029-10.502), *P* = 0.045) were risk factors for WRF. Propensity score analysis showed trends towards an increased risk for WRF in each cutoff value for the difference in PEEP: −5 cmH_2_O (OR 0.389 (95% CI 0.084-1.799), *P* = 0.229), 0 cmH_2_O (OR 2.222 (95% CI 0.755-6.540), *P* = 0.150), and 5 cmH_2_O (OR 3.277 (95% CI 0.940-11.425), *P* = 0.065).

Conclusions: This study revealed that the difference in PEEP between days 1 and 4 was positively associated with WRF. However, a significant cutoff value for the difference in PEEP was not determined.

## Introduction

Acute kidney injury (AKI) is a clinical pathology characterized by worsening renal function (WRF). It has been reported that high central venous pressure (CVP), a hemodynamic parameter, could be a risk factor for AKI in critically ill patients [1]. It is thought that elevated CVP causes renal dysfunction by impeding renal venous return and induces renal interstitial edema. Some reports have suggested that high CVP is associated with AKI in various pathologies, including after cardiopulmonary bypass surgery [2] and in sepsis [3]. Moreover, researchers have reported that positive end-expiratory pressure (PEEP) and/or mechanical positive pressure ventilation could be risk factors for AKI due to a physiological increase in CVP. Therefore, several reports have investigated whether mechanical ventilation and/or PEEP are potential risk factors for WRF and thus cause AKI. One meta-analysis showed a relationship between mechanical ventilation and AKI, but not between PEEP and WRF [4].

Recent studies have revealed that the postoperative intubation time was associated with AKI following cardiac surgery [5], and there were strong relationships between time-weighted PEEP and WRF, and between mechanical ventilation and WRF [6]. Moreover, Kaushik et al. reported that PEEP was associated with AKI in pediatric cases of acute respiratory distress syndrome (ARDS), which requires continuous high PEEP [7]. Therefore, it is possible that continuous high PEEP, as well as temporary high PEEP, could cause WRF in critically ill patients.

In this study, we investigated the association between PEEP and WRF in a cohort of patients with ARDS as a representative pathology that requires respiratory management using mechanical ventilation and PEEP, or continuous high PEEP.

In Japan, a nationwide prospective observational registry of ARDS, sepsis, trauma, severe soft tissue infection, and burns (FORECAST ARDS registry) was established by the Japanese Association for Acute Medicine (JAAM). Patients were registered between 2016 and 2017. We performed retrospective analyses of this registry to investigate the association between PEEP and WRF, and to determine whether continuous PEEP is a potential risk factor for WRF.

In previous studies, temporary PEEP and/or a PEEP of only 5 cmH_2_O were not identified as significant risk factors for WRF. We considered those earlier findings and sought to clarify the relationship between PEEP and WRF by focusing on patients with ARDS who received continuous high PEEP (≥5 cmH_2_O) to examine whether continuous high PEEP is a potential risk factor for WRF, using data from the JAAM FORECAST ARDS registry.

## Materials and methods

Study design

In this present study, we performed retrospective analyses of data from the nationwide, multicenter, observational JAAM FORECAST ARDS registry, which was conducted in Japan between January 2016 and March 2017. A total of 43 hospitals participated in the registry (see Acknowledgements). Patients diagnosed with acute lung injury (ALI) or ARDS according to the American-European Consensus Conference (AECC) definition [8] were registered. Anonymized patient data were entered into the online database. This registry was managed primarily at Hokkaido University Hospital. The original protocol was approved by the Ethics Committees of Hokkaido University Hospital, and at the ethics committees at each participating hospital. This sub-analysis was approved by the Ethics Committees of Mie University Hospital in accordance with the Japanese Ethical Guidelines for Medical and Biological Research Involving Human Subjects. Informed consent was obtained from all study participants.

Patients

Between January 2016 and March 2017, a total of 166 cases of ALI/ARDS according to the AECC definition were registered to the database. We retrieved data for patients aged ≥15 years whose renal Sequential Organ Failure Assessment (SOFA) score was recorded on day 1 and/or day 4.

Study outcomes and statistical analysis

The following data were retrieved from the database: age, sex, vital signs on admission (respiratory rate, heart rate, systolic blood pressure, diastolic blood pressure, Glasgow Coma Score, and body temperature), serum lactate and arterial partial pressure of oxygen/inspired oxygen concentration (P/F) ratio on admission, use of mechanical ventilation, Acute Physiology and Chronic Health Evaluation (APACHE) II score (day 1), total SOFA score (day 1), renal SOFA score (days 1 and 4), JAAM disseminated intravascular coagulation score (day 1), PEEP (days 1 and 4), 28-day mortality, in-hospital mortality, ventilator-free days, and intensive care unit-free days.

The renal SOFA score is 0: creatinine (Cre) <1.2 mg/dL, 1: Cre 1.2-1.9 mg/dL, 2: Cre 2.0-3.4 mg/dL, 3: Cre 3.5-4.9 mg/dL or urine output <500mL/day, and 4: Cre ≥5.0mg/dL or urine output <200mL/day.

The patients were divided into two groups according to whether or not they experienced WRF, which was defined as an increase in the renal SOFA score between days 1 and 4. The clinical data were compared between the two groups by using univariate and multivariable analyses. Univariate analyses were performed by the Mann-Whitney U test or Fisher’s exact test, as appropriate. Multivariable analyses were performed by using logistic regression analysis, in which the dependent variable was WRF, and the explanatory variables were age, sex (male), P/F ratio, lactate, APACHE II score, mechanical ventilation management, PEEP on day 1, and the difference in PEEP between days 1 and 4.

Propensity score analysis was performed by taking into account the variables age, sex (male), P/F ratio, lactate, APACHE II score, mechanical ventilation, and PEEP on day 1 using the inverse probability of the treatment-weighting (IPTW) method in order to compare the risk of WRF in subgroups of patients divided by cutoff values for the difference in PEEP between days 1 and 4 (the PEEP on day 4 minus the PEEP on day 1).

In all analyses, a P-value of <0.05 was considered statistically significant. All statistical analyses without the propensity score analysis were performed with SPSS version 25.0 (IBM, Armonk, NY, USA). Propensity score analysis with the IPTW method was performed with R software version 4.0.1 (R Foundation).

## Results

**Figure 1:**
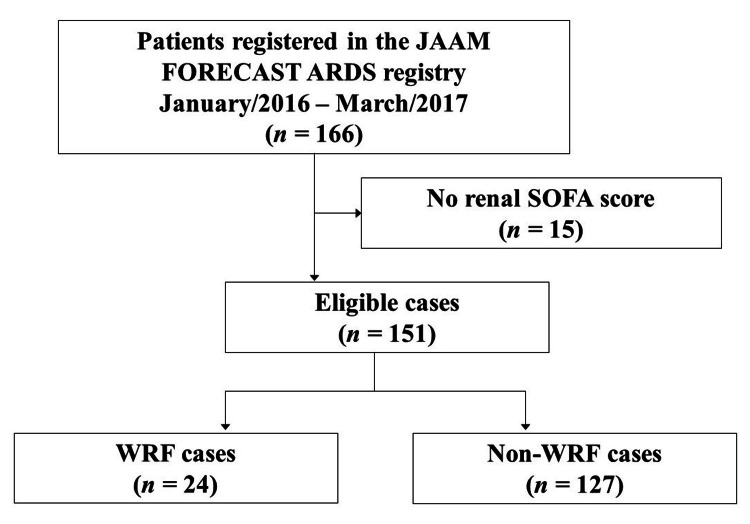
Patient disposition A total of 151 patients were considered eligible after excluding patients without a renal SOFA score. The eligible patients were divided into two groups: those with worsening or non-worsening renal function. ARDS: acute respiratory distress syndrome; JAAM: Japanese Association of Acute Medicine; SOFA: Sequential Organ Failure Assessment; WRF: worsening renal function

The registry comprised 166 patients, of which 151 met the inclusion criteria (i.e., age ≥ 15 years and renal SOFA score recorded on days 1 and 4). Thus, the inclusion rate was 91% (151/166; Figure 1).

Table 1 shows the characteristics of the WRF (n = 24) and non-WRF (n = 127) groups. Age, sex, vital signs, lactate levels on admission, and the outcomes did not differ significantly between the two groups. However, the APACHE II score and the renal SOFA score on day 4 were significantly different between the two groups.

**Table 1:** Characteristics of the WRF and non-WRF groups Values are median (interquartile range) or n (%) WRF: worsening renal function; RR: respiratory rate; HR: heart rate; sBP: systolic blood pressure; dBP: diastolic blood pressure; BT: body temperature; P/F ratio: arterial partial pressure of oxygen/inspired oxygen concentration; APACHE: Acute Physiology and Chronic Health Evaluation; SOFA: Sequential Organ Failure Assessment; JAAM: Japanese Association for Acute Medicine; DIC: disseminated intravascular coagulation; PEEP: positive end-expiratory pressure; VFD: ventilator-free day; ICUFD: intensive care unit free day

Variables	WRF	Non-WRF	P value
	(n = 24)	(n = 127)	
Age (years)	76 (67-79)	72 (58-79)	0.311
Male	19 (79%)	78 (61%)	0.109
RR (bpm)	25 (22-33)	24 (18-30)	0.389
HR (bpm)	104 (83-122)	110 (95-130)	0.402
sBP (mmHg)	115 (99-141)	109 (90-125)	0.092
dBP (mmHg)	54 (44-65)	60 (49-72)	0.367
BT (°C)	37.6 (36.5-38.3)	37.4 (36.6-38.4)	0.917
Lactate (mmoL/L)	5.9 (4.8-7.2)	6.6 (5.4-7.9)	0.382
P/F ratio	118 (83-187)	122 (78-170)	0.923
Mechanical ventilation	22 (92%)	103 (81%)	0.255
APACHE II score	25 (23-33)	23 (19-28)	0.068
Total SOFA score	9 (7-13)	9 (7-13)	0.435
Renal SOFA score on day 1	0.5 (0.0-1.0)	0.0 (0.0-1.0)	0.840
Renal SOFA score on day 4	3.0 (1.0-4.0)	0.0 (0.0-0.0)	<0.001
JAAM DIC score	4.0 (2.0-6.0)	3.0 (2.0-5.0)	0.224
PEEP on day 1 (cmH_2_O)	9.5 (6.0-10.0)	10.0 (5.0-12.0)	0.844
PEEP on day 4 (cmH_2_O)	10.5 (6.5-15.8)	10.0 (7.0-12.0)	0.176
Difference (cmH_2_O)	2.0 (-1.0 to 6.0)	0.0 (-2.0 to 4.0)	0.312
28-day mortality	5 (24%)	25 (20%)	0.773
Hospital mortality	11 (46%)	44 (35%)	0.356
VFD (days)	8.5 (0.0-17.8)	9.0 (0.0-20.0)	0.941
ICUFD (days)	6.0 (0.0-16.8)	9.5 (0.0-17.8)	0.555

Table 2 shows the results of the multivariable analysis. Male sex (odd ratios (OR) 3.332 (95% confidence interval (CI) 1.043-10.640), P = 0.042) and the difference in PEEP between days 1 and 4 (OR 1.130 (95% CI 1.024-1.247), P = 0.015) showed significant positive associations with WRF.

**Table 2:** Multivariable analysis of risk factors for WRF WRF: worsening renal function; OR: odds ratio; CI: confidence interval; P/F ratio: arterial partial pressure of oxygen/inspired oxygen concentration; APACHE: Acute Physiology and Chronic Health Evaluation; PEEP: positive end-expiratory pressure

Variables	OR (95% CI)	P value
Age	1.032 (0.993-1.074)	0.109
Male	3.287 (1.029-10.502)	0.045
P/F ratio	1.002 (0.993-1.011)	0.654
Lactate	1.058 (0.801-1.397)	0.690
APACHE II score	1.051 (0.985-1.121)	0.132
Mechanical ventilation	2.171 (0.341-13.814)	0.412
PEEP on day 1	1.050 (0.942-1.171)	0.380
Difference in PEEP	1.123 (1.017-1.240)	0.022

Table 3 shows the results of the propensity score analysis using the IPTW method, which was performed to determine the risk of WRF among cutoff values for the difference in PEEP between days 1 and 4. The risk of WRF was not significantly associated with cutoff values of −5 cmH_2_O (OR 0.389 (95% CI 0.084-1.799), P = 0.229), 0 cmH_2_O (OR 2.222 (95% CI 0.755-6.540), P = 0.150), and 5 cmH_2_O (OR 3.277 (95% CI 0.940-11.425), P = 0.065).

**Table 3:** Proportions of patients with WRF according to cutoff values for the difference in PEEP between days 1 and 4, using propensity score analysis with the IPTW method The propensity score analysis incorporated the following variables: age, sex (male), P/F ratio, lactate, APACHE II score, mechanical ventilation, and PEEP on day 1. WRF: worsening renal function; PEEP: positive end-expiratory pressure; OR: odds ratio; CI: confidence interval; P/F ratio: arterial partial pressure of oxygen/inspired oxygen concentration; APACHE: Acute Physiology and Chronic Health Evaluation

Variables	Cutoff	n	WRF	OR (95% CI)	P value
PEEP difference (cmH_2_O)	>−5	134	20 (15%)	0.389 (0.084-1.799)	0.229
(from days 1 to 4)	≤−5	17	4 (24%)		
	>0	72	13 (18%)	2.222 (0.755-6.540)	0.150
	≤0	79	11 (14%)		
	>5	33	7 (21%)	3.277 (0.940-11.425)	0.065
	≤5	118	17 (14%)		

## Discussion

In the present study, the results of the multivariable analysis indicated that a difference in PEEP between days 1 and 4 is a potential risk factor for WRF. In particular, the difference in PEEP was positively correlated with WRF (OR 1.123 (95% CI 1.017-1.240) P = 0.022; WRF: 2.0 vs non-WRF: 0.0).

Although PEEP has traditionally been considered a risk factor for WRF, few studies have proven this association. Akker et al. did not detect an association between PEEP and AKI in a meta-analysis, although 25% of patients in the high PEEP group had a PEEP of <10 cmH_2_O compared with >70% of patients in the low PEEP group [4]. Therefore, that review might not reflect current PEEP management.

Heringlake et al. reported, in post-cardiac surgery patients, 62.3% of patients who required mechanical ventilation for 16 h developed AKI, compared with 17% of patients who required mechanical ventilation for 4 h (the PEEP values were not reported) [5]. Geri et al. reported a significant association between PEEP and WRF (relative risk ratio 1.36 (95% CI 1.16-1.6), P < 0.001) in a critical care population, in which 25%-35% developed WRF (the initial and maintenance PEEP values were not reported) [6]. Using the same database, Leite et al. reported that PEEP was significantly associated with severe AKI (initial PEEP, AKI vs non-AKI: 9.4 vs 6.9 cmH_2_O, P < 0.001; the duration of PEEP was not reported) [9]. The results of those studies indicate that important factors underlying the association between PEEP and WRF might be a high initial PEEP (~10 cmH_2_O) and the duration of PEEP.

In this study, the median PEEP on day 1 was ~10 cmH_2_O in both groups (median PEEP on day 1, WRF vs non-WRF: 9.5 vs 10.0 cmH_2_O, P = 0.844), and hardly changed on day 4 in both groups (median PEEP on day 4, WRF vs non-WRF: 10.5 vs 10.0 cmH_2_O, P = 0.176). Actually, 38% of WRF cases had a difference in PEEP ≥5 cmH_2_O and 24% of non-WRF, and 13% of WRF cases had a difference in PEEP ≥10 cmH_2_O and 7% of non-WRF. Therefore, this study comprised a population of patients with a high initial PEEP (~10 cmH_2_O), which was maintained for three days, but an increase in PEEP could be related to WRF (median difference in PEEP between days 1 and 4, WRF vs non-WRF: 2.0 vs 0.0, P = 0.312, multivariable OR 1.123 (95% CI 1.017-1.240), P = 0.022). To confirm whether the difference in PEEP was a risk factor for WRF, propensity score analysis was performed for cutoff values of the difference in PEEP difference. Although a significant cutoff value was not detected, higher cutoff values for the difference in PEEP nearly reached statistical significance.

Regarding current respiratory management of ARDS, Ottolina et al. reported different frequencies of AKI in patients with different PEEP values (frequency of AKI: 16% vs 38% vs 59%, for PEEP values of 9.6 vs 12.0 vs 14.7 cmH_2_O, respectively) in patients with ARDS due to COVID-19 infection as a representative pathology [10]. Valk et al., using a matched cohort, reported a higher incidence of AKI (frequency of AKI: 55.6% vs 45.9% for PEEP values of 14.3 vs 12.0 cmH_2_O, respectively) [11]. Barragan et al. reported that PEEP was higher in AKI than in non-AKI patients (AKI vs non-AKI: 12 vs 8 cmH_2_O) [12]. Although these studies focused on patients with COVID-19, the results suggest that current respiratory management using high PEEP could cause WRF and/or AKI.

In summary, although PEEP management was traditionally considered to be associated with WRF, clinical studies have not confirmed this to be a clinically significant problem. However, these concerns may be renewed with current PEEP management strategies in which PEEP exceeds 10 cmH_2_O for several days. In this study, PEEP exceeding 10 cmH_2_O for three days could be a risk factor for WRF, and an increase in PEEP may be more harmful. Further studies are needed to understand the pathology and assess whether avoiding continuous high PEEP is clinically useful or not.

This study has some limitations. First, though the registry comprised a nationwide cohort, the study was performed retrospectively, which could introduce some bias. Second, PEEP was only recorded on days 1 and 4 and was not recorded routinely between these days. Third, renal outcomes were assessed in terms of the renal SOFA score, which may lead to some bias. Fourth, although the multivariable analysis demonstrated an association between the difference in PEEP and WRF, this association was not confirmed in the propensity score analysis, meaning the cutoff values for PEEP as a risk for WRF are unclear.

## Conclusions

The results of this nationwide, Japanese, multicenter cohort study revealed that continuous high PEEP for ARDS could be a risk factor for WRF. However, further studies would be needed, which contain more renewed definitions of ARDS cases, groups showed more differences of PEEP, and other definitions of WRF for outcomes.
